# Therapeutic potential of extracellular vesicles derived from cardiac progenitor cells in rodent models of chemotherapy-induced cardiomyopathy

**DOI:** 10.3389/fcvm.2023.1206279

**Published:** 2023-07-07

**Authors:** Manon Desgres, Bruna Lima Correa, Lorena Petrusca, Gwennhael Autret, Chloé Pezzana, Céline Marigny, Chloé Guillas, Valérie Bellamy, José Vilar, Marie-Cécile Perier, Florent Dingli, Damarys Loew, Camille Humbert, Jérôme Larghero, Guillaume Churlaud, Nisa Renault, Pierre Croisille, Albert Hagège, Jean-Sébastien Silvestre, Philippe Menasché

**Affiliations:** ^1^ Université Paris Cité, Inserm, PARCC, Paris, France; ^2^ Université de Lyon, INSA, Université Claude Bernard Lyon 1, UJM-Saint-Etienne, CNRS UMR 5520, INSERM U1206, CREATIS, Saint-Etienne, France; ^3^ Plateforme Imageries du Vivant, Université Paris Cité, UFR de médecine, Paris, France; ^4^ Institut Curie, PSL Research University, Centre de Recherche, Curie CoreTech Mass Spectrometry Proteomics, Paris, France; ^5^ MEARY Cell and Gene Therapy Center, AP-HP, Hôpital Saint-Louis, Paris, France; ^6^ Université Paris Cité, AP-HP, Hôpital Saint-Louis, MEARY Cell and Gene Therapy Center, Hôpital Saint Louis, INSERM CIC-BT CBT501, Paris, France; ^7^ FUJIFILM Cellular Dynamics, Inc., Madison, WI, United States; ^8^ Department of Cardiology, AP-HP, Hôpital Européen Georges Pompidou, Paris, France; ^9^ Department of Cardiovascular Surgery, AP-HP, Hôpital Européen Georges Pompidou, Paris, France

**Keywords:** cardiovascular progenitor, extracellular vesicles, chemotherapy-induced cardiomyopathy, cardiac strain, cardio-oncology, regenerative medicine

## Abstract

**Background:**

Current treatments of chemotherapy-induced cardiomyopathy (CCM) are of limited efficacy. We assessed whether repeated intravenous injections of human extracellular vesicles from cardiac progenitor cells (EV-CPC) could represent a new therapeutic option and whether EV manufacturing according to a Good Manufacturing Practices (GMP)-compatible process did not impair their bioactivity.

**Methods:**

Immuno-competent mice received intra-peritoneal injections (IP) of doxorubicin (DOX) (4 mg/kg each; cumulative dose: 12 mg/kg) and were then intravenously (IV) injected three times with EV-CPC (total dose: 30 billion). Cardiac function was assessed 9–11 weeks later by cardiac magnetic resonance imaging (CMR) using strain as the primary end point. Then, immuno-competent rats received 5 IP injections of DOX (3 mg/kg each; cumulative dose 15 mg/kg) followed by 3 equal IV injections of GMP-EV (total dose: 100 billion). Cardiac function was assessed by two dimensional-echocardiography.

**Results:**

In the chronic mouse model of CCM, DOX + placebo-injected hearts incurred a significant decline in basal (global, epi- and endocardial) circumferential strain compared with sham DOX-untreated mice (*p* = 0.043, *p* = 0.042, *p* = 0.048 respectively) while EV-CPC preserved these indices. Global longitudinal strain followed a similar pattern. In the rat model, IV injections of GMP-EV also preserved left ventricular end-systolic and end-diastolic volumes compared with untreated controls.

**Conclusions:**

Intravenously-injected extracellular vesicles derived from CPC have cardio-protective effects which may make them an attractive user-friendly option for the treatment of CCM.

## Introduction

Cancer survivors who have been treated with anthracyclines are at risk of developing a left ventricular (LV) dysfunction (1), even several years after the end of their cancer treatment (2). The incidence of this chemotherapy-induced cardiomyopathy (CCM) can be as high as 48% for those who have received the highest cumulative anthracycline doses and is exacerbated by the presence of other cardiovascular risk factors (3). These patients require careful monitoring, sometimes a reduction in dosing regimens or heart failure preventive therapies, whose benefit on improving patient outcomes still remains variable, especially after an anthracycline-based regimen (4, 5). Hence, new therapeutic options are eagerly awaited. Cell therapy has emerged as one of them, as documented by the protective effects of mesenchymal stromal cells (MSCs) or embryonic stem cells on post-doxorubicin cardiomyopathy (6, 7). Although the recently completed SENECA trial [(7), NCT02509156], which entailed a single catheter-based endomyocardial injection of allogeneic MSCs, did not meet its primary end point, it also reported some encouraging efficacy signals.

Although cell transplantation has been initially conceived as a “replacement” therapy whereby grafted cells would structurally engraft in the myocardium and replace dysfunctional cardiomyocytes, there is now mounting evidence that cells exert their beneficial effects through paracrine signaling mediated by the cellular secretome whose content is largely packaged in proteolipid bilayered extracellular vesicles (EV). These particles are able to transfer their biologically active cargo (composed of proteins, lipids and coding and non-coding nucleic acids) into recipient cells through membrane fusion, endocytosis or ligand/receptor binding, thereby harnessing endogenous repair pathways in the target cells (8, 9). The role of these EV in mitigating post-chemotherapy cardiac dysfunction has been reported for EV derived from cardiovascular progenitor cells (CPCs) (8) and MSCs (9). Similarly, the secretome of human amniotic fluid-derived multipotent stem cells has been shown to limit doxorubicin-induced toxicity on human CPCs (10). While the precise mechanism of action of the cells still remains elusive, what is known from the identity of their secreted biomolecules leads to speculate that they could act on specific chemo-triggered abnormalities which primarily include DNA damage, oxidative and energetic stress leading to inflammation, extracellular matrix remodeling and defects in heart contractility, all of which can contribute to LV dysfunction (1, 11, 12).

The present study was designed to assess the effects of EV isolated from CPCs in two rodent models of post-doxorubicin cardiomyopathy. The parental cell source, CPC, was selected on the basis of previous studies from our laboratory (13) and others (8) showing that EV derived from CPCs can mitigate inflammation and adverse remodeling in models of acute myocardial infarction (MI) and post-MI chronic heart failure. As both events are also pathological hallmarks of anthracycline-induced cardiomyopathy (11, 12), we hypothesized that EV derived from CPCs (EV-CPC) could also positively counteract them and thus contribute to improve heart contractility*.* We also tested whether a process compliant with a clinical-grade manufacturing scale-up and Good Manufacturing Practices (GMP)-compatible methods would not impair EV bioactivity.

## Methods

### Vesiculation and EV isolation

#### EV-CPC

CPCs differentiated from human induced pluripotent stem cells (iCell® CPC, FCDI) were thawed and plated on fibronectin pre-coated culture plates (CellBIND® HYPERFlask®, Corning) in an enriched medium (William's E Medium supplemented by Cocktail B from Hepatocyte Maintenance Supplement Pack, human bFGF and Gentamicin) as previously described (14). EV-CPC were then isolated from the conditioned medium after 2 days of serum-free and growth factor-free culture (only William's E Medium and gentamicin), clarified by a series of centrifugations (400 *g*, 10 min; 2,000 *g*, 30 min; room temperature), purified by ultrafiltration (centrifugal filter unit with vertical membrane, Amicon Ultra-15, PLTK, 30 kDa, Merck) and cryo-preserved at −80°C.

#### GMP-EV

Human iPSC-derived CPC were produced at the Innovation Facility for Advanced Cell Therapy (iFACT, FUJIFILM Cellular Dynamics, Inc, Madison, USA). CPC generation was performed in a GMP suite using a novel differentiation process with GMP-compatible methods, materials and reagents at a Phase 1 clinical manufacturing scale (prototype-GMP-CPC) (Ravel et al., manuscript in preparation, patent pending). These CPC were then cryo-preserved and shipped to the MEARY Cell and Gene Therapy Center, AP-HP Paris, France, where they were thawed and processed for vesiculation. Following collection of the conditioned medium, EV were isolated using tangential flow filtration according to GMP-compatible procedures (Patent pending).

### EV-CPC characterization

EV-CPC were characterized with regard to the number of particles (by Nanoparticle Tracking Analysis [NTA]; (NanoSight LM-10, Malvern), morphology (by immuno-gold labelling and cryo-transmission electron microscopy [CryoTEM (13)], surface marker expression (by flow cytometry using the MACSPlex Exosome Kit, Miltenyi Biotech) and protein content (Bicinchoninic Acid Assay).

### Proteomic analysis of EV-CPC

The proteomic cargo of EV-CPC was processed and analyzed as extensively described in Supplemental Material. The mass spectrometry proteomics data have been deposited to the ProteomeXchange Consortium via the PRIDE partner repository with the dataset identifier PXD022129 (Username: reviewer_pxd022129@ebi.ac.uk, Password: Z8DFhSCS).

### Rodent models of CCM, administration of EV and functional cardiac evaluations

**Figure 1:**
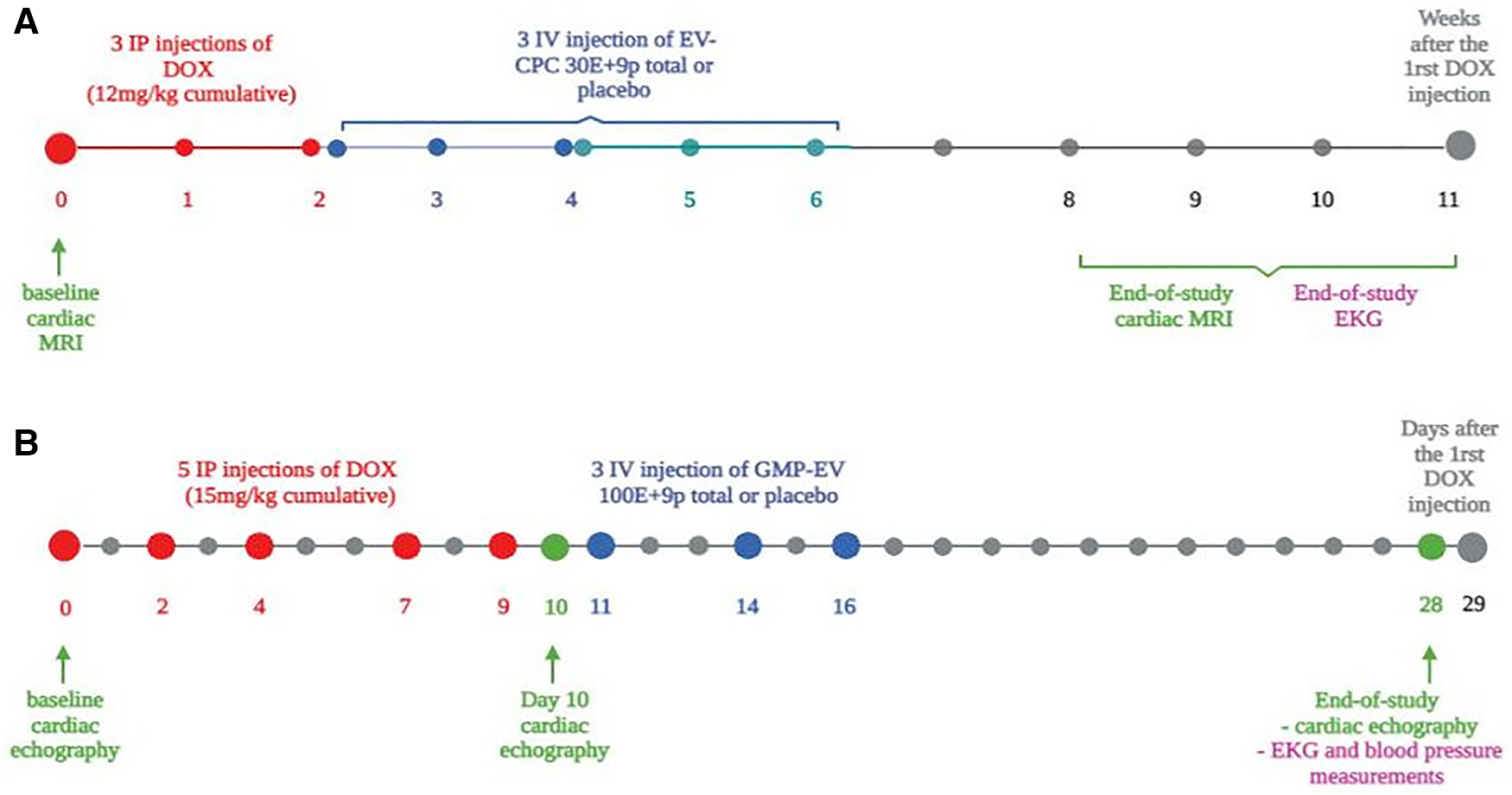
Rodent CCM model experimental protocols. (**A**) Mice were subjected to 3 weekly intraperitoneal injections of doxorubicin (DOX) (cumulative dose = 12 mg/kg). Sham-operated mice underwent isotonic buffer injections without DOX treatment.Three equal doses of EV-CPC (total dose of 30E+9 particles) were given intravenously (IV) by the retro-orbital sinus (*n* = 30) according to 3 slightly different time frames: starting 15 days after the last Dox injection and performed every 4-5 days (one series) or every 3-4 days (one series) or starting the day after the last Dox injection and performed every 4-5 days (one series). A placebo control group underwent injections of isotonic buffer according to the same timing and delivery protocol. Cardiac MRI and EKG measurements were performed to assess cardiac function. Baseline values correspond to the functional measurements which were taken in 5 healthy mice at the onset of the protocol. (**B**) Wistar female rats received 5 IP injections of DOX (3mg/kg each; total cumulative dose 15mg/kg) followed by 3 intravenous injections of GMP-EV (100E+9/injection, one every 2/3 days; *n* = 12). Control rats were placebo-injected with saline (*n* = 11) or sham-treated (no DOX; *n* = 6). Cardiac echocardiography measurements were acquired at baseline before DOX treatment, between DOX and GMP-EV treatment at day 10 (D10), and 29 days after the first DOX injection (end-of-study). At this time, EKG and blood pressure measurements were also performed. Rats were sacrificed at end-of-study, 29 days after the beginning of DOX treatment. In order to avoid selection bias, DOX animals were randomized into the 2 different treatment groups relative to their clinical status, mostly their weight loss percentage compared to baseline. MRI, magnetic resonance imaging; IP, intraperitoneal; IV, intravenous; EKG, electrocardiogram.

All procedures were approved by the Institutional Ethics Committee from Paris University (project #15616 and #33136) and complied with European legislation (European Commission Directive 2010/63/EU) on animal care. They are illustrated in Figures 1A,B and detailed in Supplemental Material.

In brief, immune-competent BALB/c mice received 3 weekly intra-peritoneal (IP) injections of doxorubicin (DOX; 4 mg/kg each; cumulative dose: 12 mg/kg), were then intravenously (IV) injected three times with EV (total dose: 30 billion) over 2 weeks and finally assessed 9–11 weeks later by cardiac magnetic resonance imaging (FLASH cine sequences on a 4.7 T preclinical Bruker scanner) (Figure 1A). Absolute values of Circumferential Strain (CS) and Longitudinal Strain (LS) measures (Tissue-tracking module, Circle Cardiovascular Imaging software, Biomedical Imaging Research Laboratory CREATIS, Lyon, France) are expressed both as a percentage and in percentage change (%Δ) from mean baseline values set at 100%. These baseline values correspond to the functional measurements taken at the onset of the protocol in 5 healthy mice.

Then, Wistar rats received 5 IP injections of DOX (3 mg/kg each; cumulative dose 15 mg/kg) followed by 3 equal IV injections of GMP-EV (total dose: 100 billion), one every 2 days (Figure 1B). Cardiac function was assessed by two dimensional-echocardiography (parasternal long axis views in B-mode using the single-plane area-length method, VEVO Lab) at baseline before DOX treatment, between DOX and the first GMP-EV treatment at day 10 (D10) and 29 days after the first DOX injection at the end of the study.

In order to avoid selection bias, DOX-treated animals (both mice and rats) were allocated to the EV-CPC or GMP-EV and control groups on the basis of their clinical status at the end of drug treatments, mostly their weight loss from baseline so as to ensure the comparability of measurements. Myocardial contour tracing was processed by the same operator, blinded to the treatment group and echocardiographic measurements were double-checked by a senior cardiologist.

### qRT-PCR from explanted mouse hearts

RNA was isolated from mouse hearts and processed for qRT-PCR as presented in Supplementary Figure S4.

### Statistics

Statistical analyses are detailed in Supplemental Material. To estimate the effect size of EV treatment, the Cohen's d index for unequal variance was calculated and interpreted according to a commonly accepted stratification where small, medium, large and very large effect sizes are considered for values of 0.2, 0.5, 0.8 and 1.3, respectively.

## Results

### EV-CPC characterization

**Figure 2:**
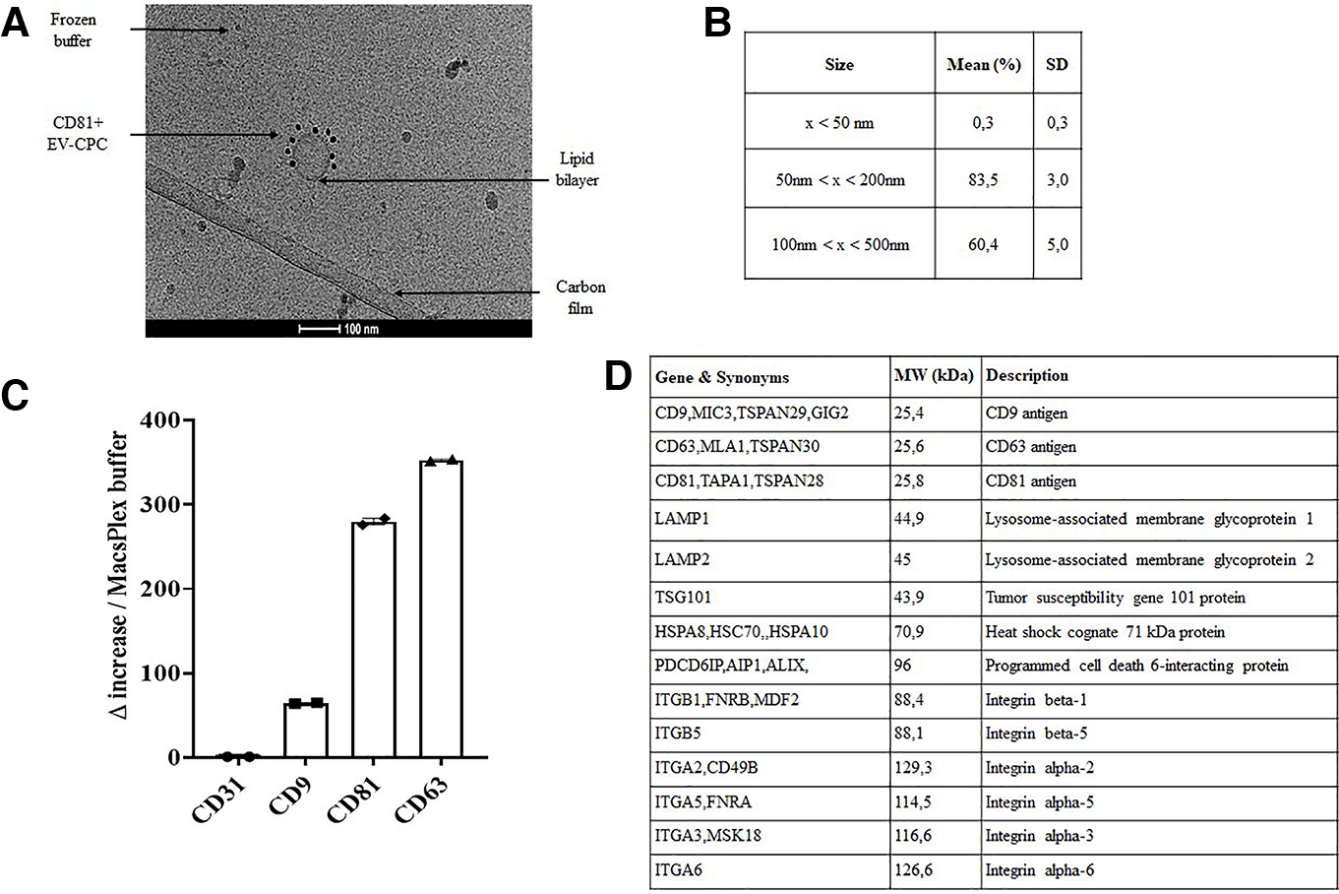
EV-CPC characterization. (**A**) Cryo-TEM resolution of EV-CPC lipid bilayer membranes. Scale bar = 100 μm. (**B**) Size distribution of isolated particles by NTA analysis measured on 5 different preparations of EV-CPC. (**C**) FACS detection of tetraspanin expression on EV-CPC by MACSPlex analysis compared to a negative control, i.e., the CD31 endothelial marker (in technical duplicates; median +/− IQR). (**D**) Extract from the absolute quantification of proteomic analysis. SD: standard deviation; NTA: Nanoparticle Tracking Analysis; Cryo-TEM: immuno-gold labelling and cryo-transmission electron microscopy; CD: cluster of differentiation; MW: molecular weight.

Following the MISEV 2018 guidelines (15), the CPC secretome was characterized by multiple and complementary methods which demonstrated that intracellular material was encapsulated in particles exhibiting a lipid bilayer membrane, the smallest of which (<100 nm) were densely CD81 positive (Figure 2A). As shown in Figure 2B, particles ranged in size from 50 nm to 450 nm (with almost 85% of them with a size below 200 nm) and expressed key surface markers (LAMP1/2, CD9, CD63, CD81) (Figures 2C,D). The presence of cytosolic proteins with lipid or protein binding capabilities (TSG101, HSC70, ALIX and ITGB1/5 and ITGA2/3/5/6) was also confirmed. The median protein content in the final EV-CPC suspension was 140 µg/ml and the median number of particles per CPC was 25,000.

### EV-CPC improve heart contractility in CCM mice

To validate the ability of our DOX regimen to induce a cardiomyopathy, end-study data recorded in DOX-injected mice were compared with baseline values collected at the onset of the protocol in 5 healthy mice. Compared with these normal mice, DOX resulted in a significant impairment of Global Longitudinal Strain (GLS; −13.35 vs. −14.80, *p* ≤ 0.046). Likewise, compared with sham-operated (DOX-free, saline-injected) mice, those injected with DOX incurred declines in diastolic mass and ejection fraction, an increase in LV volumes (Supplementary Figures S1A–E) and a QTc interval lengthening with a decreased cardiac output (without changes in heart rate) (Supplementary Figures S1F–J). Similar trends were observed when papillary muscles were included in the segmentation of the inner diameter (Supplementary Figures S2A–F). However, although DOX also caused liver damage (Supplementary Figure S3A) treated mice did not exhibit pulmonary edema (Supplementary Figure S3B) or extensive fibrosis, as assessed by histology (Supplementary Figures S3C,D) or qRT-PCR (Supplementary Figures S3E-G), thereby suggesting that our protocol captured an early stage of ventricular dysfunction.

**Figure 3:**
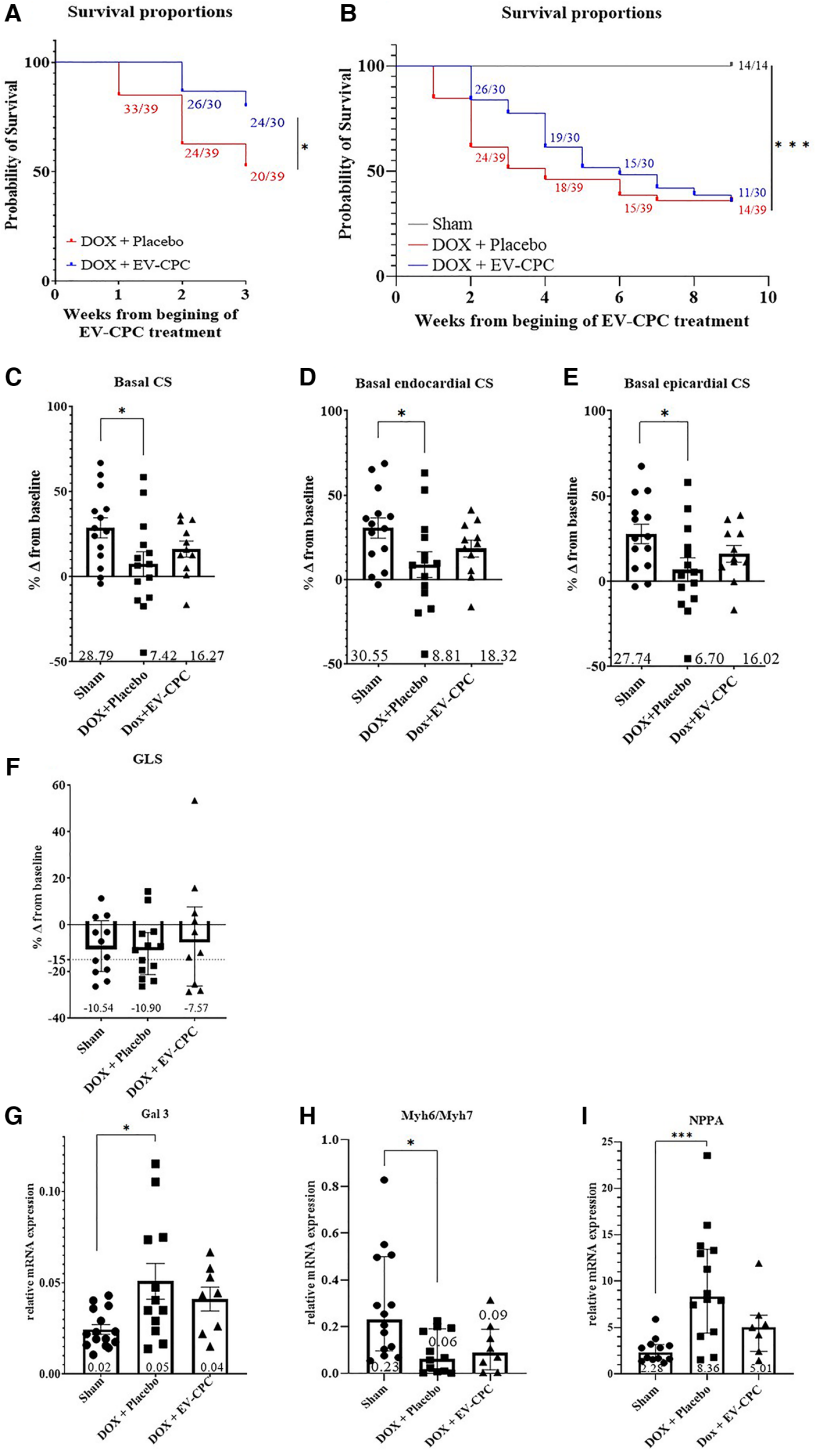
EV-CPC treatment improves heart contractility of CCM mice and decrease adverse remodeling. Survival Kaplan Meier curves with Log rank analysis test performed (**A**) at 3 weeks after the beginning of DOX treatment and (**B**) at end-of-study. MRI evaluation of cardiac parameters (C-F): (**C**) CS measured by MRI (Mean + / − SEM) at the basal global, (**D**) basal endocardial and (**E**) basal epicardial levels (Sham: *n* = 14; DOX+Placebo: *n* = 14, DOX+EV-CPC: *n* = 11); and (**F**) GLS (Median +/− IQR) as a percent change from baseline (some mice, two in sham group and one in each EV or placebo group, could not be included into GLS datasets due to unavailable 2-chamber views in MRI: Sham: *n* = 14; DOX+Placebo: *n* = 14, DOX+EV-CPC: *n* = 11). Relative mRNA expression in mouse hearts of Gal3 (G; Mean +/− SEM), Myh6/Myh7 (H; Median +/− IQR) and NPPA (I; Median +/− IQR): these data integrated a supplemental series of Sham and DOX+placebo animals exclusively used for PCR assessment. *p ≤ 0.05; ***p ≤ 0.001. CS, circumferential strain (%); GLS, Global longitudinal strain; Gal 3, galectin 3; Myh6/Myh7 ratio, myosin heavy chain α/myosin heavy chain *β*; NPPA, Atrial natriuretic peptide.

EV-CPC significantly improved the early survival of mice compared with placebo-injected controls (Figure 3A) even though curves subsequently tended to merge, likely because of DOX-induced disease progression and the related clinical deterioration (Figure 3B). Furthermore, while DOX + placebo hearts incurred a significant decline in basal (global, endo- and epicardial) CS (Figures S3C–E) compared with sham mice (*p* = 0.043, *p* = 0.048, *p* = 0.042 respectively), EV-CPC preserved these indices which did not significantly differ from those of the sham group. Similar patterns of changes were seen for GLS (Figure 3F). Furthermore, if one sets at −15% the threshold for a clinically relevant decline in GLS predictive of LV dysfunction (16, 17), there were twice as many placebo-injected mice in this subgroup than EV-CPC-treated ones (Effect size Cohen's d index of 0.4). Finally, while DOX-treated placebo-injected hearts demonstrated an upregulation of genes involved in fibrosis (Galectin 3) along with fetal and pathological gene expression associated with heart failure (decrease in the myosin heavy chain Myh6/Myh7 ratio, increase in NPPA) compared with DOX-free sham-operated mice (Figures 3G–I), EV-CPC treatment partially prevented these changes conducive to maladaptive remodeling and LV systolic dysfunction (effect size Cohen's d index of 0.4, 0.2 and 0.9 for Galectin 3, Myh6/Myh7 and NPPA respectively).

### GMP-EV preserve left ventricular volumes in CCM rats

To confirm the effects of CPC-derived EV and assess them in a more translational perspective, we repeated the protocol but in a different animal species and with EV which had been manufactured according to GMP-compatible procedures (Figure 1B).

**Figure 4:**
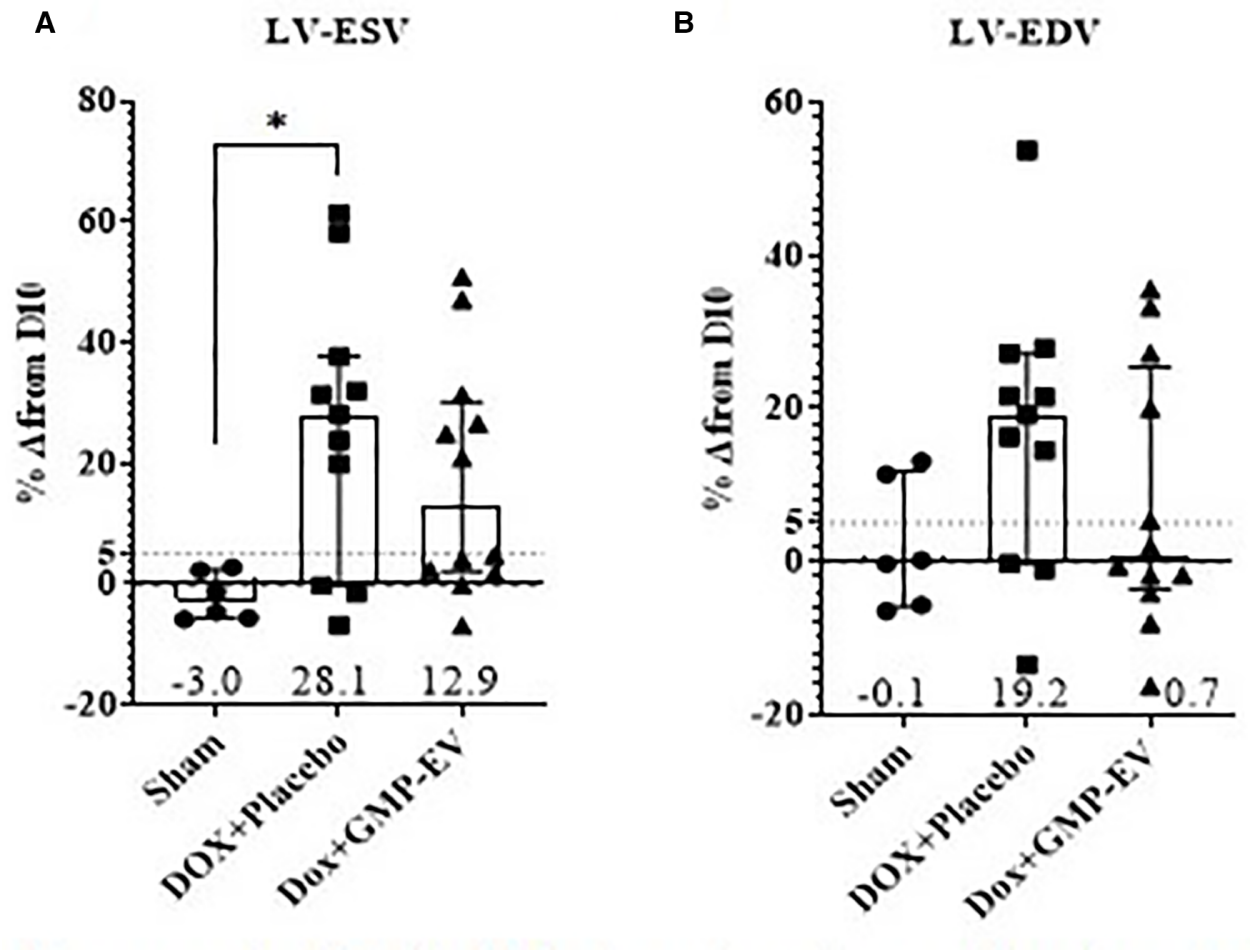
GMP-EV treatment moderates left ventricular dilatation upon rats doxorubicin exposure. (**A**) LVESV and (**B**) LVEDV measured by echocardiography and expressed as a percent change (Median+/−IQR) from day 10 (post-DOX administration). Sham: *n* = 6; DOX+Placebo: *n* = 11, DOX+GMP-EV: *n* = 12. *p ≤ 0.05; (Kruskal Wallis with Dunn's correction test). LV-ESV/LV-EDV, left ventricular-systolic/diastolic function.

Among the 27 initially included rats, 4 died before the end-of-study time point, thereby leaving 6 sham-operated, 11 placebo- and 12 EV-injected animals available for the final analysis. At this time point, our DOX infusion protocol fostered LV dysfunction characterized by a decrease in LVEF (Supplementary Figure S5A), an impaired systolic elastance (assessed on the systolic blood pressure/LVESV ratio) without a noticeable elevation of blood pressure (Supplementary Figures S5B,C) and a slower LV-depolarization (Supplementary Figure S5D). To adjust for the variability in the response of rats to chemotherapy, end-study data are presented as percent changes from those recorded at day 10, i.e., the end of DOX treatment, and allocation of rats to GMP-EV of placebo injections was then made as to ensure their comparability at this time point. While LVESV volumes were significantly increased in placebo-injected hearts compared with DOX-untreated sham (*p* = 0.033), they were preserved by GMP-EV injections (effect size Cohen's d index of 0.4) (Figures 4A,B). Likewise, the percentages of “responder” rats which did not increase their LVEDV volumes by more than 5% from their post-DOX pre-treatment values were 58% (7 out of 12) vs. 28% (3 out of 11) in GMP-EV- and placebo-injected hearts, respectively (effect size Cohen's d index of 0.5).

## Discussion

So far, none of the drugs commonly used for preventing CCM have shown unequivocal efficacy. Among potential new treatments, stem cells have been successfully tested in animal models (6, 18). The clinical experience is still more limited despite some hints of efficacy in the recent SENECA trial (7). At the same time, the increasing recognition that cells act primarily through paracrine signaling, largely mediated by EV, has led to consider using these particles instead of their parental cells since, from a translational perspective, they feature clinically relevant advantages such as stability under cryo-storage, off-the-shelf availability, lack of immunogenicity (depending on the parental cell source) and manufacturing processes more akin to those of pharmaceutics.

In this study, we used a molecular weight cut-off of the ultrafiltration and tangential flow filtration membranes which allowed to harvest a blend of cell-released biomolecules extending beyond the exclusive collection of EV because superior outcomes have been reported after delivery of almost the full cellular secretome compared with highly purified exosomal or protein fractions (19, 20). Thus, although the word EV has been used throughout the manuscript, the tested product would be better qualified as an EV-enriched secretome. Of note, in our experiments, equally positive outcomes were observed with EV-CPC (of research-grade, for mouse experiments) and GMP-EV (for rat experiments), thereby providing the reassuring observation that a process at clinical manufacturing scale and using GMP-compatible methods does not seem to impair EV bioactivity.

Our choice of collecting this secretome from CPCs was dictated by the finding that better outcomes have been reported when EV are secreted by cells which belong to the same lineage as those of the tissue targeted for repair (21–23) and, furthermore, are at an early stage of differentiation which endows them with a higher secretory profile (14, 19, 22). This concept of lineage matching has also been demonstrated in models of lung fibrosis (24) or thrombo-embolic stroke (25).

While the nature of the secreting cells is one of the factors of a successful outcome, another equally important pre-requisite to a sustained therapeutic effect is likely the repeated administration of cells or their secreted products (26, 27)*.* This implies the route of delivery to be noninvasive and user-friendly which highlights the interest of the intravenous approach. At first glance, such an approach may look counterintuitive in view of biodistribution studies which have shown that EV delivered intravenously are predominantly sequestered in the lungs, spleen and liver, with few of them reaching the heart (28–30). Nevertheless, several experimental studies have documented the cardio-protective effects of intravenously infused EV [reviewed in (27)] and these data are indirectly endorsed by the positive outcomes of clinical trials which have tested the intravenous delivery of cells (31, 32) which share the same biodistribution patterns as their EV progeny. Of note, the benefits of systemically delivered EV have also been reported in preclinical models of chronic kidney disease, lung fibrosis, hepatic ischemia-reperfusion injury and traumatic brain injury, to name a few, thereby supporting a general mechanism of organ cross-talk. The link between remotely trapped EV (or cells) and a beneficial cardiac effect remains incompletely settled but seems to primarily involve a systemic regulation of the immune response, particularly through a shift of the phenotype of endogenous monocytes/macrophages towards a pro-reparative pattern, as demonstrated in brain (25) or lung (33) injury models; by travelling through the bloodstream, these reprogrammed host immune cells would then act as secondary mediators conveying the EV protective effects to the target organ (34–36). This “bioreactor hypothesis” (35) is supported by the direct tracking of labelled vesicles trafficking in blood from the pulmonary capillary lumen to myocardial tissue (36). The justification of using the intravenous route is reinforced by a meta-analysis of the effects of EV in preclinical models of myocardial infarction which failed to identify the route of delivery (intramyocardial or intravenous) as a predictor of a successful outcome (37). Furthermore, one cannot exclude that in our experiments cardiac homing may have been facilitated by using EV derived from CPCs which may partly display the same repertoire of surface receptors as the target cells (29).

The pathophysiology of anthracycline-induced cardiomyopathy seems to primarily involve oxidative and DNA damages and mitochondrial dysfunction (1, 11, 12). That these mechanisms were, at least partly, handled by EV is supported by (1) an improved early survival of DOX + EV-CPC mice compared to placebo controls (which might have been prolonged by additional EV-CPC injections), (2) a reduced cardiac functional impairment in animals treated with EV, (3) the better maintenance of ATP levels upon exposure of doxorubicin-stressed human cardiomyocytes to EV *in vitro* (38) and (4) the consistency between the nature of EV-CPC protein cargo and changes in gene expression observed in EV-CPC treated hearts (38) which points to a protective effect of EV-CPC against altered energy metabolism and cardiomyocyte dysfunction, both of which are characteristic of anthracycline toxicity. These regulatory changes are actually in line with the finding that iPSC-derived cardiomyocytes generated from patients of the SENECA trial and exposed *in vitro* to DOX demonstrated an improved viability mediated by large mitochondria-enriched EV-CPC (39). Put together, these changes translated into the ability of EV to preserve LV function and particularly strain which is a sensitive predictor of patient outcomes (40, 41). In particular, CS has been reported as a relevant measurement for long-term follow up (42).

## Limitations

Although we adapted our *in vivo* protocols from previously published reports (43, 44), we recognize that both the severity of clinical conditions and the variability in inter-individual animal responses inherent in this model limited sample sizes and made challenging to find a trade-off between an acceptable mortality (whose the higher incidence in mice might have been exacerbated by their male sex while rats were females) (45, 46) and the development of a left ventricular dysfunction leaving some room for demonstration of a therapeutic effect. For this reason, common *p*-value-based statistical methods were complemented by the assessment of effect sizes. It could also be argued that the DOX doses administered were low compared with the human ones (36 mg/m^2^ for human homothetic doses) (47). The reason is that the IP route in rodents results in a bolus-type of delivery which differs from the slow intravenous infusions practiced in the clinics, which likely accounted for the rapid clinical deterioration of mice and the presence of large ascites in rats, thereby precluding a longer-term follow-up. We cannot either exclude that the expected therapeutic effect of EV was weakened by their clearance by the murine immune system given the xenogenic setting of our protocol (48).

## Conclusions

Despite these limitations, the study provides a proof of principle that repeated IV administrations of EV can be effective to improve DOX-induced cardiomyopathy at its earliest stages of development. In the future, this therapeutic benefit might be further optimized by improving EV cardiac targeting by surface changes engineered to express ligands specific for inflamed myocardium (49). Finally, it is important to stress that in the perspective of an upcoming phase 1 clinical trial, we have been able to duplicate, in a rat model of CCM, the benefits of EV-CPC following their preparation compatible with GMP standards. Taking together, these results justify further exploring this path as a means of preventing and/or reversing energetic stress and cardiac remodeling induced by anthracyclines before overt cardiac dysfunction. This strategy would have the additional advantage of avoiding cessation or dose limitation of anti-cancer treatments which may jeopardize their effectiveness against the causative disease.

## Data Availability

The datasets presented in this study can be found in online repositories. The names of the repository/repositories and accession number(s) can be found below: ProteomeXchange Consortium via the PRIDE partner repository with the dataset identifier PXD022129 (Username: reviewer_pxd022129@ebi.ac.uk, Password: Z8DFhSCS).
