# Supplementary material for: Therapeutic potential of extracellular vesicles derived from cardiac progenitor cells in rodent models of chemotherapy-induced cardiomyopathy
Source: Front Cardiovasc Med. 2023 Jul 7;10:1206279. doi: 10.3389/fcvm.2023.1206279 (PMC10360184; doi:10.3389/fcvm.2023.1206279)
Supplement: Supplementary file 1 [file Datasheet1.pdf]

Supplemental Material

Mouse CCM model (Figure 1A, Figure 3 A-B)

Seventy-six male BALB/c mice (BALB/cJRj; 9 to 12-week-old; Janvier Labs) were subjected to 3 weekly intraperitoneal (IP) injections of DOX (doxorubicin ACCORD, 2 mg/ml) for a total cumulative dose of 12 mg/kg (Figure 1A). Among these 76 DOX-injected mice, 7 died over the course of DOX injections before receiving any treatment, thus letting 69 survivors. In order to avoid selection bias and ensure the comparability of outcome measurements, these DOX-treated mice were then allocated to the EV-CPC or control groups on the basis of their clinical status at the end of anthracycline treatment, mostly their weight loss from baseline. Three equal doses of EV-CPC (total dose of 30E+9 particles) were given intravenously (IV) (by the retro-orbital sinus; n=30) over 2 weeks. A placebo control group underwent injections of isotonic buffer according to the same timing and delivery protocol (n=39). Forty-four of the DOX-injected mice (EV and placebo) were subsequently euthanized during the study period because they had met the humane endpoints established for the study, leaving 14 placebo-injected and 11 EV-CPC treated mice available for the end-of-study time point.

Fourteen additional mice were sham-operated and underwent isotonic buffer (Sodium Chloride Solution 0.9%, Fresenius Kabi) IP injections without DOX treatment. Mice were sacrificed 9-11 weeks after the beginning of treatments.

Functional cardiac evaluation and MRI parameters analysis (Figure 3 C-F; Supplemental Figures 1-2)

The primary end point was LV function assessed by cardiac magnetic resonance imaging (CMR) performed on a 4.7T preclinical scanner (Biospec 47/40 USR, Bruker) with a transmit volume coil combined with a four-channel phased array surface coil specific for the mouse heart. Mice were induced with 4% isoflurane in air and then anesthetized with 2% isoflurane, based on a respiration rate of 80 breaths/min. The temperature of mice was stabilized at 37 °C using a heating blanket. A video of the short-axis of the heart was acquired with intragate FLASH cine sequences: 8 slices of 1-mm thickness acquired sequentially with TR/TE=7.2/2.7ms and a flip angle of 18 °, planar resolution of 117 µm, 16 cardiac cycle images, oversampling of 150 with an acquisition time of 2 min 18 s per slice. Videos were also acquired in a long-axis slice with the same parameters. For some mice, a four-chamber slice was also acquired. In addition, mice underwent EKG analyses which were performed on the on EKG Analysis Module (LabChart ® & PowerLab®) according to the manufacturer's instructions. Subdermal needles detect PQRST onset, amplitude and interval to assess heart function with species-specific detection settings. The module's Beat Averaging feature reduces noise and movement artifact.

Circle Cardiovascular Imaging software (cvi 42 version 5.13) packages were used for blinded off-line quantitative assessment on CINE acquisitions by the Biomedical Imaging Research Laboratory CREATIS (Lyon, France). Global function was assessed by manually tracing the left ventricular (LV) inner endocardial and outer epicardial borders in the short axis at end-diastole and end-systole. Papillary muscles were systematically excluded from the segmentation of the inner diameter by using a software feature. After validation, the software calculates the LV volume-based parameters, including EndSystolic Volume (ESV in ml), EndDiastolic Volume (EDV in ml), myocardial mass (g) and Ejection Fraction (EF [%] defined as (EDV-ESV)/EDV × 100) measured in all datasets. Tissue-tracking module was used to

determine LV global longitudinal strain (GLS) in all animals with a regional (endocardial and epicardial) segmentation performed in 2 (and 4-chamber views when possible) at end-diastole and then automatically propagated throughout the whole cardiac cycle. When available, GLS was calculated from the average of GLS values obtained in 2 and 4-chambers views. Otherwise, only the 2-chambers view was analyzed. Tracking accuracy was visually inspected and corrections were made by the operator to the initial contour (if necessary) before re-applying the tracking algorithm. For the circumferential strain (CS) evaluation, short axis sections were analyzed at the basal level (the most basal section including a complete visualization of the circular myocardium at end-systole). Regional CS evaluations (epicardial and endocardial values) were also analyzed.

Absolute values of Circumferential Strain (CS) and Longitudinal Strain (LS) measures are expressed both as a percentage and in percentage change (%Δ) from mean baseline values set at 100%. These baseline values correspond to the functional measurements which were taken in 5 healthy mice at the onset of the protocol.

Papillary muscles were also included in the segmentation of the inner diameter by using a software feature and data are presented in Supplemental Figure 2.

Proteomic analysis (Figure 2D)

Sample Preparation

Cryopreserved cardiac progenitor cells (CPC) differentiated from human iPSC (iCell® Cardiac Progenitor Cells, KDR^+/PDGFR-α^+/C-KIT^-, Cellular Dynamics International) were thawed and plated at a density of 78,000 cells/cm^2 and 5,000 cells/cm^2 respectively, on fibronectin-coated flasks (Roche Applied Sciences) and cultured 4 days at 7% CO_2 and 37 °C in serum-free media according to the proliferation protocol recommended by the manufacturer: William's E medium (Life Technologies) with Cocktail B from hepatocyte maintenance supplement pack (Life Technologies), 25 µg/mL gentamicin (Life Technologies) and bFGF, 1 µg/mL (Miltenyi). The particle concentration in the media prior to exposure to cells (virgin media) was $4x10^8$ particles/mL. The media was refreshed on day 2, the flasks were washed 4 times with phosphate-buffered saline to eliminate all serum contaminants and cells were cultured for 2 additional days in only William's E medium and gentamicin. At day 4, the conditioned media (containing the EV secreted over the previous 48 hours) was collected for EV isolation. Conditioned media were pre-cleared by centrifugation for 6 minutes at 1,200 g and EV-CPC were pelleted by ultrafiltration (Amicon) using a molecular weight cut-off membrane of 3 kD.
Ten µg of each sample were dried and solubilized in 20 µL 8 M urea, 200 mM ammonium bicarbonate and then reduced in 5 mM dithiothreitol, pH 8 with vortexing at 37°C for 1 h. After cooling to room temperature, cysteines were alkylated by adding 10 mM iodoacetamide for 30 min in the dark. After diluting to 1 M urea with 200 mM ammonium bicarbonate pH 8.0, samples were digested with 1 µg trypsine/LysC (Promega), with vortexing at 37 °C overnight. Samples were then loaded onto homemade C18 StageTips for desalting. Peptides were eluted using 40/60 MeCN/H_2O + 0.1% formic acid, vacuum concentrated to dryness and reconstituted in injection buffer (0.3% TFA) before nano-LC-MS/MS analysis.

LC-MS/MS Analysis

Liquid chromatography (LC) was performed with an RSLCnano system (Ultimate 3000, Thermo Scientific) coupled to an Orbitrap Exploris 480 mass spectrometer (Thermo Scientific). Peptides were first trapped on a C18 column (75 µm inner diameter × 2 cm; nanoViper Acclaim PepMap™ 100, Thermo Scientific) with buffer A (2/98 MeCN/H_2O

in 0.1% formic acid) at a flow rate of 3.0 µL/min over 4 min. Separation was then performed on a 50 cm x 75 µm C18 column (nanoViper Acclaim PepMap™ RSLC, 2 µm, 100Å, Thermo Scientific) regulated to a temperature of 40°C with a linear gradient of 3% to 29% buffer B (100% MeCN in 0.1% formic acid) at a flow rate of 300 nL/min over 91 min. MS full scans were performed in the ultrahigh-field Orbitrap mass analyzer in ranges *m/z* 375–1500 with a resolution of 120 000 at *m/z* 200. The top 20 most intense ions were subjected to Orbitrap for further fragmentation via high-energy collision dissociation (HCD) activation and a resolution of 15,000 with the AGC target set to 100%. We selected ions with charge state from 2+ to 6+ for screening. Normalized collision energy (NCE) was set at 30 and the dynamic exclusion at 40 s.

Data analysis

For identification, data were searched against the *Homo Sapiens* Swiss-Prot database (UP000005640) and a databank of the common contaminants using Sequest-HT through proteome discoverer (version 2.4). Enzyme specificity was set to trypsin and a maximum of two miss cleavages sites were allowed. Oxidized methionine, Methionine-loss, Methionine-loss-Acetyl and N-terminal acetylation were set as variable modifications. Carbamidomethylation of cysteins were set as a fixed modification. Maximum allowed mass deviation was set to 10 ppm for monoisotopic precursor ions and 0.02 Da for MS/MS peaks. The resulting files were further processed using myProMS v3.9 (1). FDR calculation used Percolator (2) and was set to 1% at the peptide level for the whole study. Peptides Extracted Ion Chromatograms (XICs) were extracted by using MassChroQ version 2.2.17 (3) and a condition-specific alignment was performed for re-extracting missing XICs across replicates.

Also, protein molar and mass percentage were estimated by using Top 3 (4) (including proteins with less than 3 peptides) as the Protein Quantification Index and the direct proportionality model (5).

The mass spectrometry proteomics data have been deposited to the ProteomeXchange Consortium via the PRIDE (6) partner repository with the dataset identifier PXD022129 (Username: reviewer_pxd022129@ebi.ac.uk,Password: Z8DFhSCS).

RNA isolation and qRT-PCR of explanted mouse hearts (Figure 3 G-I, Supplemental Figure 3 D-G and Supplemental Figure 4)

After sacrifice, explanted mice hearts were washed in phosphate-buffered saline, the atria were removed and hearts were dissected into 2 parts: the apex (1/4 of the heart volume) was directly frozen in liquid nitrogen vapor for transcriptomic analysis and the remaining part (3/4 of the heart volume) was submerged in Optimal Cutting Temperature compound (OCT, Tissue-Tek; Sakura Finetek) before freezing.

Total RNA was extracted from mouse heart tissue lysed with Polytron (IKA Ultra Turrax T25) in TRIzol™ Reagent (Invitrogen) according to the manufacturer's instructions. The RNA concentration and purity were assessed by NanoDrop One® Spectrophotometry (ThermoFisher Scientific).

Primers were designed with Oligo 7 software (Molecular Biology Insights). One-hundred nanograms of total RNA were used to be reverse-transcribed (QuantiTect Reverse Transcription Kit, Qiagen). SYBR® Green reagents (Meridian Bioscience) were prepared and distributed in 96-well plates manually or with the PIPETMAN® (Pipetmax 268 Gilson). PCR was performed

on StepOnePlus System (ThermoFisher Scientific) and gene expression was analyzed on StepOne™ Software and compared to Hypoxanthine Guanine Phosphoribosyl Transferase (HPRT) constitutive gene expression. The list of primers is indicated in Supplemental Figure 4.

Morphological parameters (Supplemental Figure 3 A-B)

Mice and rats' lungs, livers and spleen were harvested to be weighted. Lungs were also weighted after 24 h in a 60 °C oven. Right legs were harvested and tibia lengths were measured with a caliper after the leg tissues had been lysed (Tris 50 mM, EDTA 5 mM; SDS 0.5%, NaCl 0.9% 100 mM, proteinase K, 550 ug/mL) overnight in a 37 °C oven (SupplementalFigure 3A and B).

Interstitial fibrosis evaluation (Supplemental Figure 3C-D)

Frozen, OCT-embedded heart samples were cryo-sectioned into 7-µm thin sections. Sirius Red staining was used to visualize and quantify interstitial fibrosis. Frozen tissue slices were immersed in ethanol 80 % (10 min) and then rehydrated in water. Coloration was done in a Sirius red bath (10 g / 100 mL of picric acid - 16 min) and rinsed in water, ethanol 95 %, ethanol 100%, and xylene baths (3 min each). Eukitt mounting medium was used. Images were acquired on Nanozoomer XR (Hamamatsu Photonics) slide scanner and were manually quantified using Image J software (at least 3 pictures on 4 tissue slices taken at 3 different levels of the heart were analyzed). The investigator was blinded to treatment groups.

Rat CCM model and echocardiographic parameters analysis (Figur 1B, Figure 3, Supplemental Figure 5)

Twenty seven Wistar female rats (RjHan:W; 8-week-old; Janvier Labs) received 5 IP injections of DOX (3 mg/kg each; total cumulative dose 15mg/kg) followed by 3 equal IV injections (by caudal vein) of GMP-EV (total dose of 100E+9 particles, one every 2 days) (Figure 1B). Control rats were placebo-injected with isotonic buffer or sham-operated (no DOX). In order to avoid selection bias and ensure the comparability of outcome measurements DOX-treated rats were allocated to the GMP-EV and control groups on the basis of their clinical status at the end of drug treatments, mostly their weight loss from baseline. Cardiac function assessment was performed (under 1.5-2% isoflurane anesthesia) with two dimensional-echocardiography (Visual Sonics 2100 ultrasound system (FUJIFILM)) equipped with a 20-MHz transducer probe. Data were acquired at baseline before DOX treatment, between DOX and GMP-EV treatment at day 10 (D10), and 29 days after the first DOX injection at the end of the study (D29). Body temperature, respiratory and heart rates were controlled throughout the acquisitions. Echocardiographic values of Left-Ventricular-End-Systolic Volume (LVESV) and Left-Ventricular-End-Diastolic Volume (LVEDV) were calculated from parasternal long axis views in B-mode (VEVO Lab) using the single-plane area-length method. Outlines were traced by the same operator, blinded to the treatment group and measurements were double-checked by a senior cardiologist. At the end of the study, rats were lightly anesthetized (1.5% isoflurane) and electrocardiograms were performed with the EKG Analysis Module for LabChart ® & PowerLab®, as previously described. Blood pressure measurements were also performed with a noninvasive Volume Pressure Recording (VPR) technology (CODA® High Throughput System, Kent Corporation), which measures the physiological characteristics of the returning blood flow after occlusive inflation of a tail cuff.

Statistics

All data were submitted to GraphPad Prism Outlier tool (ROUT; Q = 10%) before creating graphs and calculating statistics. Normality of each variable distribution was tested by D'Agostino & Pearson and Shapiro-Wilk tests. A Mann Whitney U test was used to compare 2 groups of non-parametric distribution. A Kruskal-Wallis test by ranks was used to compare 3 or more independent experimental groups of non-parametric distribution. Comparisons between groups were then performed by using Dunn's multiple comparisons test. Reciprocally, when variables presented a normal distribution, an unpaired t-test was used to compare 2 groups and a one-way ANOVA was used to compare 3 or more independent experimental groups corrected with Tuckey method for pairwise group comparisons. To compare survival curves, a Log-rank test was performed. A two-tailed p value ≤ 0.05 was considered significant. To estimate the effect size of EV treatment, Cohen's d index for unequal variance was calculated as follows (SD= Standard deviation, S=average variance):

$$Cohen's\ d = \frac{|Mean_{EV} - Mean_{Dox}|}{S} \quad where\ S = \sqrt{\frac{(SD_{EV}{}^2 + SD_{DOX}{}^2)}{2}}$$

Interpretation of this index was based on a commonly accepted stratification where small, medium, large and very large effect sizes are considered for values from 0.2, 0.5, 0.8 and 1.3, respectively. In one control rat for which the end-of-study recording could not be done, imputation of the missing data was done according to Last Observation Carried Forward (LOCF) method.

All statistical analyses were double-checked by an independent statistician.

Supplemental Material Figures

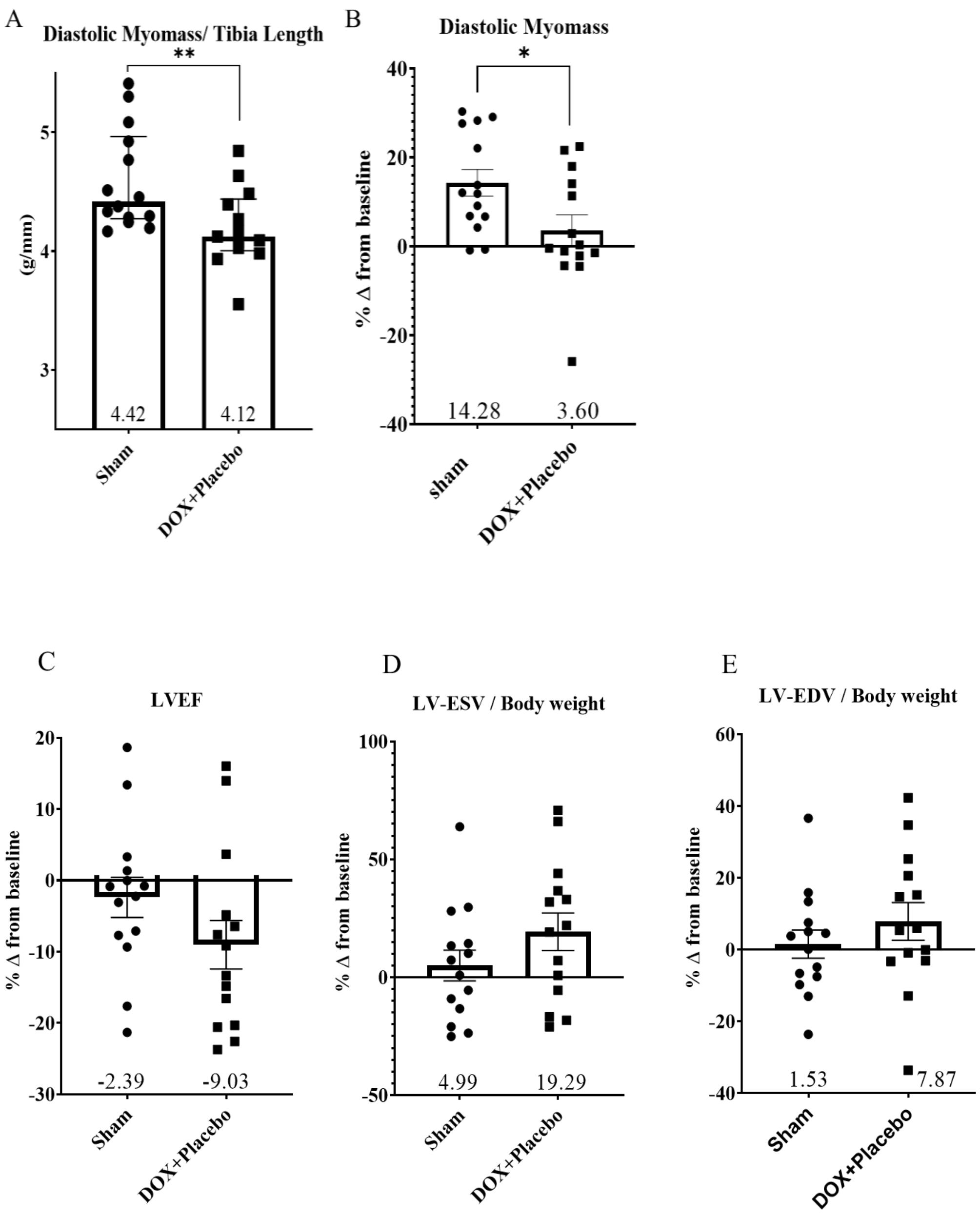
A
Diastolic Myomass/ Tibia Length
**
(g/mm)
5
4
3
4.42
4.12
Sham
DOX+Placebo
B
Diastolic Myomass
*
% Δ from baseline
40
20
0
-20
-40
14.28
3.60
sham
DOX+Placebo
C
LVEF
% Δ from baseline
20
10
0
-10
-20
-30
-2.39
-9.03
Sham
DOX+Placebo
D
LV-ESV / Body weight
% Δ from baseline
100
50
0
-50
4.99
19.29
Sham
DOX+Placebo
E
LV-EDV / Body weight
% Δ from baseline
60
40
20
0
-20
-40
1.53
7.87
Sham
DOX+Placebo

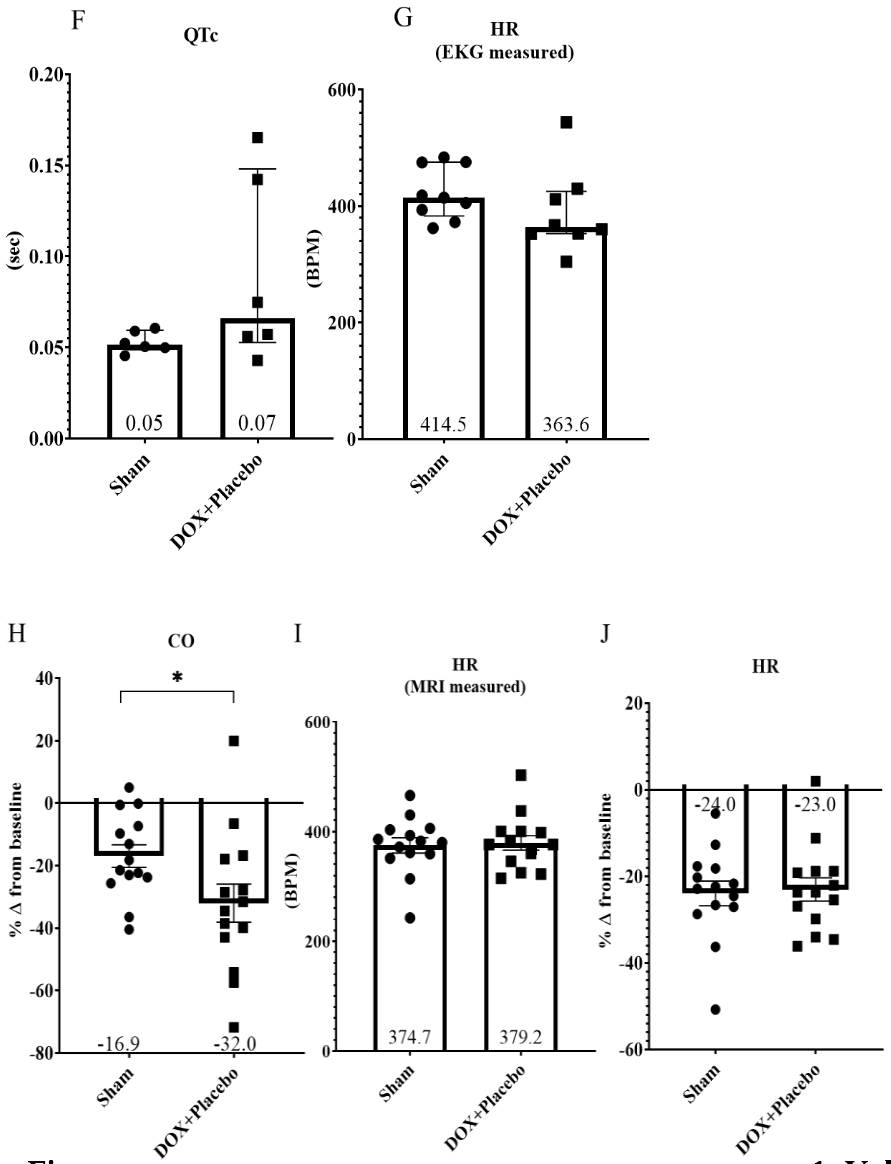

<u>Supplemental Figure 1: Validation of the mouse model of DOX-induced cardiomyopathy</u>.

(A) Diastolic myo-mass compared to tibia length (median+/-IQR) expressed in absolute values or (B) as a percent change from baseline (Mean+/-SEM); (C) LVEF as a percent change from baseline (Mean+/-SEM); (D) LVESV and (E) LVEDV normalized to body weight as percent change from baseline (Mean+/-SEM). Sham: n=14; DOX+Placebo: n=14.
Impact of DOX on electrical heart activity and cardiac output. (F) Subdermal evaluation of ventricular systole duration by electrocardiogram analysis (QT length corrected by heart rate) and (G) Heart rate (Sham: n=6; DOX: n=6) (Median+/-IQR).
MRI evaluation of cardiac parameters (Mean+/-SEM): (H) Cardiac output and (I) Heart rate in absolute values or (J) as percent change from baseline $*p \leq 0.05$; $**p \leq 0.01$; $***p \leq 0.001$. Mann Whitney test was used to compare 2 groups of non-parametric distribution; Unpaired t-test was used to compare 2 groups of parametric distribution. Baseline values refer to those recorded in healthy mice at the onset of the protocol.
Myomass: muscular diastolic mass, LVESF/LVEDV: left ventricular end-systolic/end diastolic volumes (%), LVEF: left ventricular ejection fraction (%), QTc: length of QT segment corrected to HR (sec); HR: heart rate (BPM), EKG: electrocardiogram, CO: cardiac output.

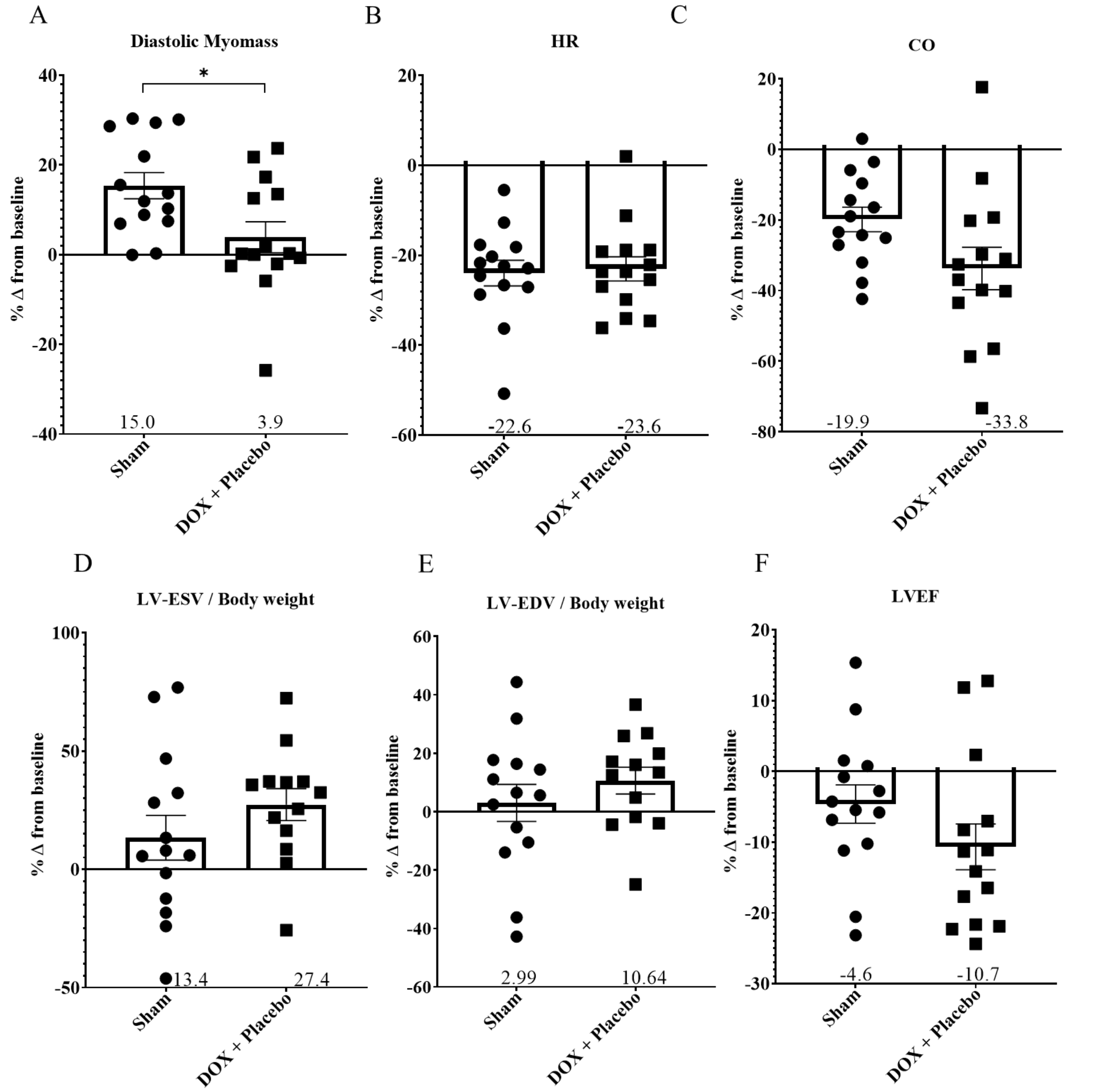

<u>Supplemental Figure 2: Volumetric cardiac parameters with inclusion of papillary muscles in the segmentation of the inner diameter</u>.

(A) Diastolic myomass as a percent change from baseline (mean+/-SEM); (B) Heart rate as a percent change from baseline (mean+/-SEM); (C) Cardiac output as a percent change from baseline; (D) and (E) LVESV and LVEDV normalized to body weight as a percent change from baseline (Mean+/-SEM) and (F) LVEF as a percent change from baseline (Mean+/-SEM) calculated according to the following formula : LVEF = ((LV-EDV - LV-ESV) / LV-EDV) × 100 (Sham: n=14; DOX+Placebo: n=14); *p≤0.05 (Mann Whitney test was used to compare 2 groups of non-parametric distribution; Unpaired t-test was used to compare 2 groups of parametric distribution). Baseline values refer to those recorded in healthy mice at the onset of the protocol.

Myomass: muscular diastolic ventricular mass, LV-ESF/LV-EDV: left ventricular systolic/diastolic volumes (%), LVEF: left ventricular ejection fraction (%).

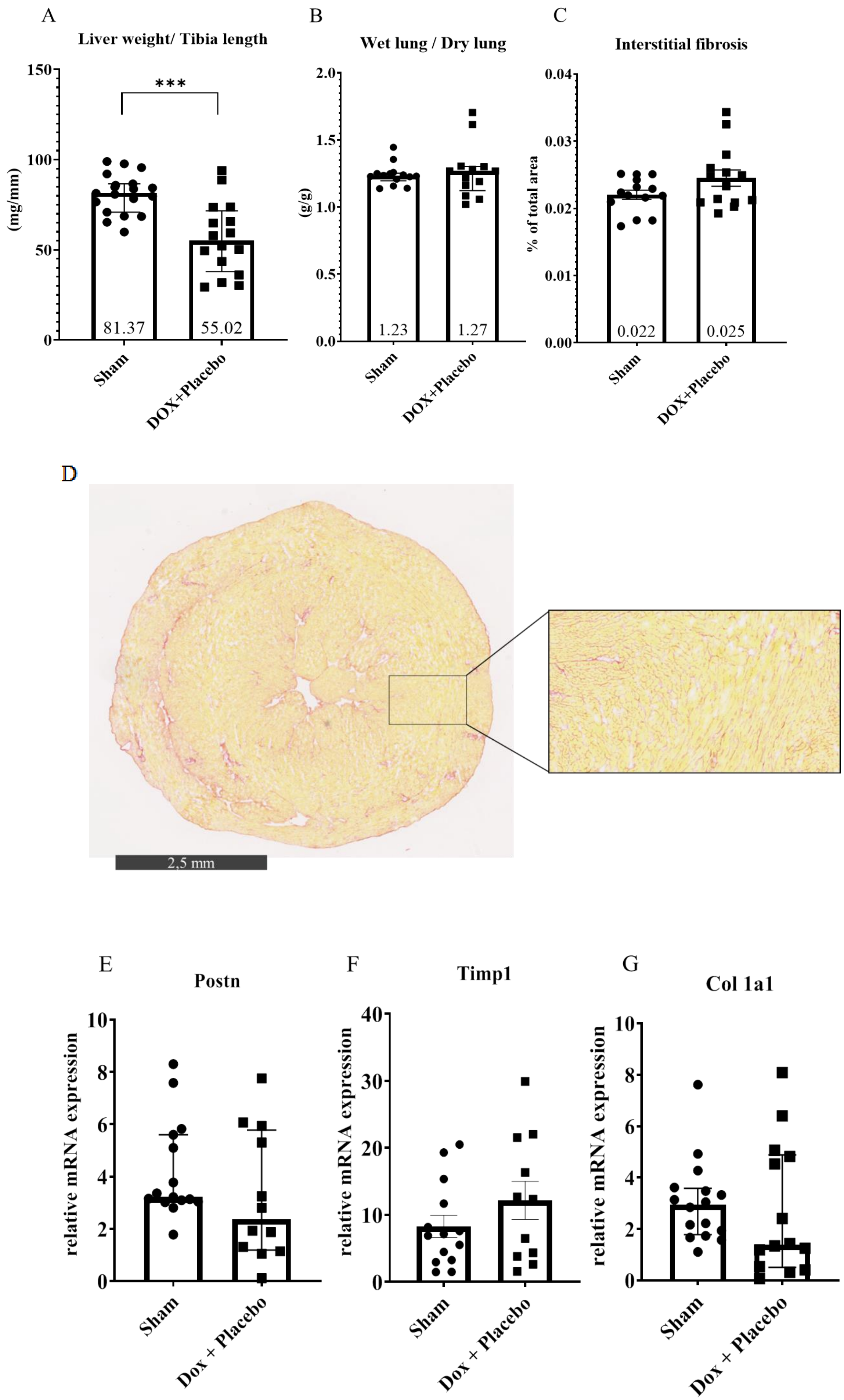
A
Liver weight/ Tibia length
***
(mg/mm)
150
100
50
0
81.37
55.02
Sham
DOX+Placebo
B
Wet lung / Dry lung
(g/g)
2.0
1.5
1.0
0.5
0.0
1.23
1.27
Sham
DOX+Placebo
C
Interstitial fibrosis
% of total area
0.04
0.03
0.02
0.01
0.00
0.022
0.025
Sham
DOX+Placebo
D
2,5 mm
E
Postn
relative mRNA expression
10
8
6
4
2
0
Sham
Dox + Placebo
F
Timp1
relative mRNA expression
40
30
20
10
0
Sham
Dox + Placebo
G
Col 1a1
relative mRNA expression
10
8
6
4
2
0
Sham
Dox + Placebo

Supplemental Figure 3 : Early stage of ventricular dysfunction preceding the occurrence of irreversible damage. (A) Liver DOX-off-target effects (Median+/-IQR), (B) lung water content (Median+/-IQR), (Sham: n=14; DOX: n=14); (C) interstitial fibrosis expressed as a percentage of the total heart area (Sirius Red) (Mean+/-SEM); (D) Cryofrozen Sirius red-stained section of a placebo-injected heart illustrating the limited degree of interstitial fibrosis (Left : 1.25X Magnification, Right : 10X Magnification); (E, F, G) Relative mRNA expression of fibrosis activated-genes compared to HPRT constitutive expression. *** $p \leq 0.001$. (Mann Whitney test was used to compare 2 groups of non-parametric distribution; Unpaired t-test was used to compare 2 groups of parametric distribution). *Timp1: metallopeptidase inhibitor 1 (Mean+/-SEM), Col1a1: Collagen Type I Alpha 1 Chain (Median+/-IQR), Postn: Periostin (Median+/-IQR).*

**gene**	**Sequence Forward (5 '=> 3')**	**Sequence Reverse (5 '=> 3')**	**annealing temperature (°C)**
NPPA	AGTGCGGTGTCCAACACAGATCTGA	GCGGCCCCTGCTTCCTCAGTC	64,1
Timp1	GGGCCGCCTAAGGAACGGAAATTTG	GGGCCCCAAGGGATCTCCAGGTG	64,5
Myh6	CACCGGGAAAATCTGAACAAGCTGA	CAGCGCACAAAGTGAGGGTGG	58,4
Myh7	AGCGCAGAGACTCCCTGCTGATT	TGCGGCGAGCCTCAGACTTCTCTA	63,9
Col1a1	TGCCCTCCTGACGCATGGCCAAGA	CCTCGGGTTTCCACGTCTCACCATT	62,8
POSTN	GCGGCAAGACAAGCGCTTTAGCA	CCGGGCTGTGTCAGGAGATCTTT	61,0
GAL 3	CCTGCCCTTGCCTGGAGGAGTCAT	GCGGGGGTTAAAGTGGAAGGCAACA	62.9
HPRT	AGCAGTACAGCCCCAAAATGGTT	CTGGCCTGTATCCAACACTTCGACA	59,2

Supplemental Figure 4: List of primers used for qRT-PCR.

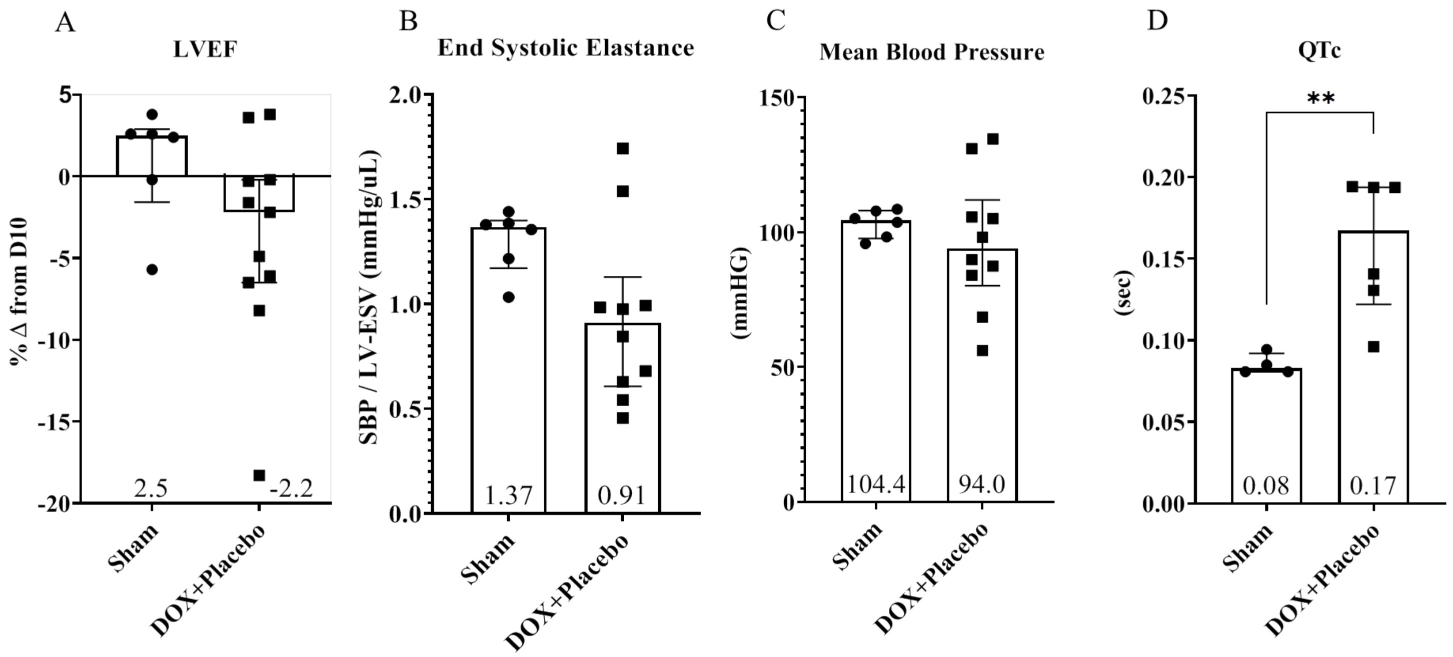

Supplemental Figure 5 Validation of the rat model of DOX-induced cardiomyopathy. (A) LVEF as a percent change from day 10 (post-DOX administration). (B) End-of-study ratio of systolic blood pressure to LV-ESV taken as a surrogate marker for ventricular contractility and (C) Mean blood pressure. (D) QT interval corrected for heart rate. (Median+/-IQR) **p≤0.001; (Mann Whitney test). *LVEF: left ventricular ejection fraction (%), QTc: length of QT segment corrected to HR (sec), DBP: diastolic blood pressure, LV-EDV: left ventricular end-diastolic volume.*

References

1. Poullet P, Carpentier S, Barillot E. myProMS, a web server for management and validation of mass spectrometry-based proteomic data. PROTEOMICS. 2007;7(15):2553–6.

2. The M, MacCoss MJ, Noble WS, Käll L. Fast and Accurate Protein False Discovery Rates on Large-Scale Proteomics Data Sets with Percolator 3.0. J Am Soc Mass Spectrom. 2016;27(11):1719–27.

3. Valot B, Langella O, Nano E, Zivy M. MassChroQ: A versatile tool for mass spectrometry quantification. PROTEOMICS. 2011;11(17):3572–7.

4. Silva JC, Gorenstein MV, Li GZ, Vissers JPC, Geromanos SJ. Absolute Quantification of Proteins by LCMSE. Molecular & Cellular Proteomics. 2006 Jan;5(1):144–56.

5. Ahrné E, Molzahn L, Glatter T, Schmidt A. Critical assessment of proteome-wide label-free absolute abundance estimation strategies. PROTEOMICS. 2013;13(17):2567–78.

6. Perez-Riverol Y, Csordas A, Bai J, Bernal-Llinares M, Hewapathirana S, Kundu DJ, et al. The PRIDE database and related tools and resources in 2019: improving support for quantification data. Nucleic Acids Res. 2019 Jan 8;47(Database issue):D442–50.
